# HB-EGF induces mitochondrial dysfunction via estrogen hypersecretion in granulosa cells dependent on cAMP-PKA-JNK/ERK-Ca^2+^-FOXO1 pathway

**DOI:** 10.7150/ijbs.69343

**Published:** 2022-02-28

**Authors:** Ji-Cheng Huang, Cui-Cui Duan, Shan Jin, Chuan-Bo Sheng, Yu-Si Wang, Zhan-Peng Yue, Bin Guo

**Affiliations:** 1 College of Veterinary Medicine, Jilin University, Changchun, P. R. China.; 2 Key Laboratory of Agro-products Processing Technology, Jilin Provincial Department of Education, Changchun University, Changchun, P. R. China.; 3 Reproductive Medical Center, Jilin University Second Hospital, Changchun, P. R. China.

**Keywords:** HB-EGF, estrogen, cAMP-PKA-JNK/ERK-Ca^2+^-FOXO1 pathway, mitochondrial dysfunction, granulosa cell

## Abstract

Polycystic ovarian syndrome (PCOS) is one of the most prevalent endocrinopathies and the leading cause of anovulatory infertility, but its pathogenesis remains elusive. Although HB-EGF is involved in ovarian cancer progression, there is still no clarity about its relevance with PCOS. The present study exhibited that abundant HB-EGF was noted in follicular fluid from PCOS women, where it might induce the granulosa cells (GCs) production of more estrogen via the elevation of CYP19A1 expression after binding to EGFR. Furthermore, HB-EGF transduced intracellular downstream cAMP-PKA signaling to promote the phosphorylation of JNK and ERK whose blockage impeded the induction of HB-EGF on estrogen secretion. Meanwhile, HB-EGF enhanced the accumulation of intracellular Ca^2+^ whose chelation by BAPTA-AM abrogated the stimulation of HB-EGF on FOXO1 along with an obvious diminishment for estrogen production. cAMP-PKA-JNK/ERK-Ca^2+^ pathway played an important role in the crosstalk between HB-EGF and FOXO1. Treatment of GCs with HB-EGF resulted in mitochondrial dysfunction as evinced by the reduction of ATP content, mtDNA copy number and mitochondrial membrane potential. Additionally, HB-EGF facilitated the opening of mitochondrial permeability transition pore via targeting BAX and raised the release of cytochrome C from mitochondria into the cytosol to trigger the apoptosis of GCs, but this effectiveness was counteracted by estrogen receptor antagonist. Collectively, HB-EGF might induce mitochondrial dysfunction and GCs apoptosis through advancing estrogen hypersecretion dependent on cAMP-PKA-JNK/ERK-Ca^2+^-FOXO1 pathway and act as a promising therapeutic target for PCOS.

## Introduction

Polycystic ovary syndrome (PCOS) is one of the most prevalent endocrinopathies with an incidence of 5 to 20% in women of childbearing age and the leading cause of anovulatory infertility followed by a heightened risk of type 2 diabetes as well as cardiovascular disease 1,2. Accumulating evidence has revealed that dysfunction of ovarian granulosa cells (GCs) may contribute to plenty of PCOS symptoms, containing menstrual irregularity, arrested follicular development and anovulation 3,4. Simultaneously, GCs from PCOS patients produced more estradiol which resulted in follicle growth arrest and its supplementation in female mice brought about anovulatory and follicular cysts 5-8. But there is limited report about the underlying mechanism of GCs dysfunction in PCOS.

Heparin-binding EGF-like growth factor (HB-EGF), a ligand of epidermal growth factor receptor (EGFR), was originally identified as a secreted glycoprotein in human macrophage medium with high affinity for heparin and exhibited an important function in female reproduction such as early embryo development, blastocyst implantation, decidualization, etc. 9,10. In rat ovary, HB-EGF was highly expressed in GCs of primordial and primary follicles followed by an apparent weakness with follicular development, becoming absent in preovulatory follicles, implying that down-regulation of HB-EGF may be essential for follicular maturation 11. Further analysis found that HB-EGF was increased in serum and peritoneal fluid of ovarian cancer patients and its blockage repressed tumour growth 12-15. However, little information is available regarding the relevance between HB-EGF and PCOS.

The present study revealed that HB-EGF was abundant in follicular fluid of PCOS patients, where it might induce the hypersecretion of estrogen and bring about mitochondrial dysfunction and apoptosis of GCs through cAMP-PKA-JNK/ERK-Ca^2+^-FOXO1 pathway dependent on EGFR.

## Materials and methods

### Collection of follicular fluid

Follicular fluids were collected from PCOS and non-PCOS women undergoing *in vitro* fertilization at the Centre of Reproductive Medicine, Second Hospital of Jilin University and their use was approved by hospital Ethics Committee concomitant with the obtainment of informed consent from all participants. Twenty PCOS patients, whose mean age was 30.83 ± 3.29 years and mean body mass index (BMI) was 28.17 ± 3.59 kg/m^2^, were diagnosed according to the Rotterdam criteria. Sixteen non-PCOS patients, who had normal ovarian morphology and regular menstrual cycles but were infertile because of tubal blockage or male factor along with mean age of 31.08 ± 3.39 years and mean BMI of 27.52 ± 2.33 kg/m^2^, were referred as control.

### GCs treatment

Human KGN ovarian GCs (Biobw) were incubated with recombinant human HB-EGF protein (rHB-EGF, 20 ng/ml, R&D Systems) for 12 h in the absence or presence of EGFR inhibitor PF299804 (500 nM, Selleck), protein kinase A (PKA) inhibitor H89 (10 μM, Selleck), c-Jun N-terminal kinase (JNK) inhibitor SP600125 (20 mM, Selleck), extracellular signal-regulated kinase (ERK) inhibitor GDC-0994 (10 μM, Selleck), intracellular calcium ion (Ca^2+^) chelator BAPTA-AM (20 μM, Selleck), forkhead box O 1 (FOXO1) inhibitor AS1842856 (10 μM, MCE), estrogen receptor antagonist ICI 182780 (0.1 μΜ, MCE) and mitochondrial permeability transition pore (mPTP) opening inhibitor ER-000444793 (2 μM, MCE). In addition, after treatment with rHB-EGF and EGFR inhibitor PF299804, cells were supplemented with cAMP analogue 8-bromoadenosine-cAMP (8-Br-cAMP, 500 μM, Sigma). PF299804, H89, SP600125, GDC-0994, BAPTA-AM, AS1842856, ICI 182780 and ER-000444793 were dissolved in DMSO, while rHB-EGF and 8-Br-cAMP were dissolved in PBS. Controls received the vehicle only.

### ELISA

Concentration of HB-EGF protein in follicular fluids was measured using a commercial ELISA kit (Cusabio). Meanwhile, after KGN GCs were treated as mentioned above, culture supernatants were gathered and then applied to determine the levels of estrogen in the light of corresponding ELISA kit (Cusabio).

### Real-time PCR

After total RNA extraction and cDNA synthetization, the expression levels of cytochrome P450 family 19 subfamily a member 1 (CYP19A1), FOXO1, Bcl2-associated X protein (BAX) and caspase 3 (CASP3) were measured by real-time PCR analysis using the corresponding primers (Table 1) as described previously 16.

### Western blotting

After extraction of total and nuclear proteins, western blotting was performed with primary antibody against JNK (1:1000, Cell Signaling Technology), phospho-JNK (1:1000, Cell Signaling Technology), ERK1/2 (1:1000, Proteintech), phospho-ERK1/2 (1:1000, Cell Signaling Technology), FOXO1 (1:1000, Proteintech), BAX (1:1000, Proteintech), CASP3 (1:1000, Proteintech), histone H3 (1:5000, Proteintech) and glyceraldehyde-3-phosphate dehydrogenase (GAPDH, 1:5000, Proteintech) as described previously 16.

### Measurement of intracellular cAMP level

After various treatments, intracellular cyclic adenosine monophosphate (cAMP) level was detected by cAMP-Glo™ Assay (Promega). Briefly, KGN GCs were incubated with Induction Buffer followed by the supplementary of cAMP-Glo™ Lysis Buffer. After addition of cAMP Detection Solution, GCs were replenished with Kinase-Glo® Reagent on the heels of the assessment of luminescence using plate-reading luminometer.

### Determination of intracellular Ca^2+^

After various treatments, KGN GCs were incubated with fluorescent probe Fluo-3 AM (5 μM, Beyotime), and then analyzed by flow cytometry to determine the level of intracellular Ca^2**+**^.

### Measurement of ATP content

After different treatments, KGN GCs were lysed and then supernatants were gathered to calculate the content of adenosine triphosphate (ATP) by the corresponding kit (Beyotime).

### Measurement of mitochondrial membrane potential (MMP)

After different treatments, KGN GCs were incubated with JC-1 fluorescent probe (Beyotime) followed by the analysis of flow cytometry or another MMP indicator TMRM (1:1000, ThermoFisher Scientific) followed by the nuclear counterstaining of Hoechst 33342 prior to the visualization in fluorescence microscope.

### Determination of mitochondrial DNA (mtDNA) copy number

After different treatments, DNA was isolated and then appraised the ratio of mtDNA/nuclear DNA (ncDNA) by real-time PCR to determine mitochondrial DNA (mtDNA) copy number as described previously 17.

### Opening of mitochondrial permeability transition pore (mPTP)

After different treatments, KGN GCs were incubated with Calcein AM (Beyotime) together with the replenishment of cobalt chloride to quench intracellular green fluorescence. Finally, cells were analyzed by flow cytometry or visualized in fluorescence microscope behind the nuclear staining with Hoechst 33342.

### Assessment of cytochrome C release

After introduction of pCytochrome C-GFP plasmid (Addgene), KGN GCs were treated as described above and then incubated with TMRM followed by the nuclear counterstaining of Hoechst 33342. Images were obtained in fluorescence microscope.

### Dual luciferase analysis

CYP19A1 promoter sequence (-123 to +41) contained FOXO1 binding site was amplified by the following primer: 5′- CTCGAGCAGACAGACCTTGCTGAGATT and 5′- AAGCTTCCTTCCTGTTTGCCTCCACG. After enzyme digestion, fragment was inserted into pGL6 luciferase reporter vector. Followed by the introduction of pGL6-CYP19A1 plasmid, GCs were treated with rHB-EGF in the absence or presence of FOXO1 inhibitor AS1842856. Afterwards, luciferase activity was measured by dual luciferase reporter gene assay kit (Beyotime). The pRL-SV40 plasmid (Beyotime) was used for data normalization.

### Cell apoptosis

KGN GCs were resuspended after trypsinization and then incubated with Annexin V-FITC (Beyotime) and propidium iodide for 20 min followed by the analysis of flow cytometry. Meanwhile, cells were lysed and then supernatants were collected to calculate the activity of CASP3 by the corresponding assay kit (Beyotime).

### Statistical analysis

All experiments were independently repeated at least three times. Significance of difference between two groups was compared by Independent-Samples T Test. The multiple comparisons were tested with one-way ANOVA with Tukey's post hoc test. Data were shown as means ± SEM. P < 0.05 was considered statistically significant.

## Results

### HB-EGF induced the hypersecretion of estrogen and GCs apoptosis via EGFR

**Figure 1:**
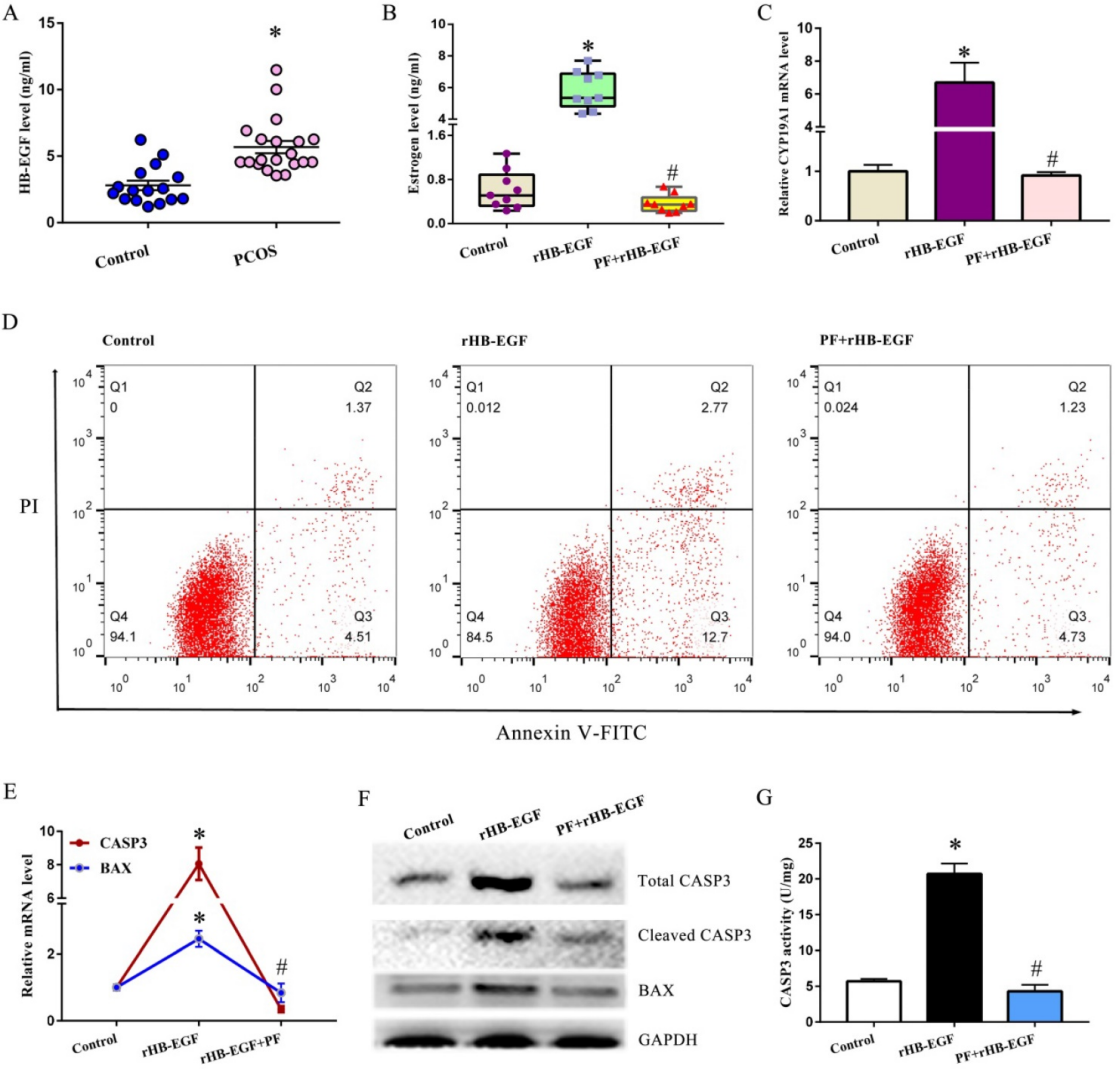
** HB-EGF induces the hypersecretion of estrogen and GCs apoptosis via EGFR. A,** HB-EGF content in follicular fluid between PCOS and non-PCOS patients. **B,** Effect of HB-EGF on estrogen secretion in the absence or presence of EGFR inhibitor PF299804. PF, PF299804. **C,** Regulation of HB-EGF on CYP19A1 expression with/without PF299804. N = 6. **D,** Effect of HB-EGF on GCs apoptosis in the existence or not of PF299804. N = 3. **E and F,** Real-time PCR and western blot analyses of CASP3 and BAX expression after treatment with rHB-EGF in the absence or presence of PF299804. N = 3. **G,** Effect of HB-EGF on CASP3 activity with/without PF299804. N = 5. * P < 0.05 versus control, # P < 0.05 versus rHB-EGF treatment.

To clarify the association between HB-EGF and PCOS, we compared the difference of HB-EGF content in follicular fluid between PCOS and non-PCOS patients and found that elevated protein level of HB-EGF was noted in follicular fluid from PCOS women (Figure 1A). Further analysis evidenced that addition of rHB-EGF caused KGN GCs production of more estrogen, but this effectiveness was blocked by PF299804 (Figure 1B), an irreversible EGFR inhibitor. Simultaneously, HB-EGF induced the expression of CYP19A1 mRNA, which was an important rate-limiting enzyme in ovarian estrogen biosynthesis, whereas replenishment of PF299804 abrogated this induction (Figure 1C).

After treatment with rHB-EGF, apoptosis rate of GCs was obviously raised (Figure 1D). Furthermore, HB-EGF raised the mRNA and protein levels of BAX and CASP3, and induced the cleavage of CASP3 concomitant with an increase for CASP3 activity. But supplementation of EGFR inhibitor PF299804 ameliorated above effectiveness conferred by HB-EGF (Figure 1E-G).

### HB-EGF activates cAMP-PKA signaling through EGFR

**Figure 2:**
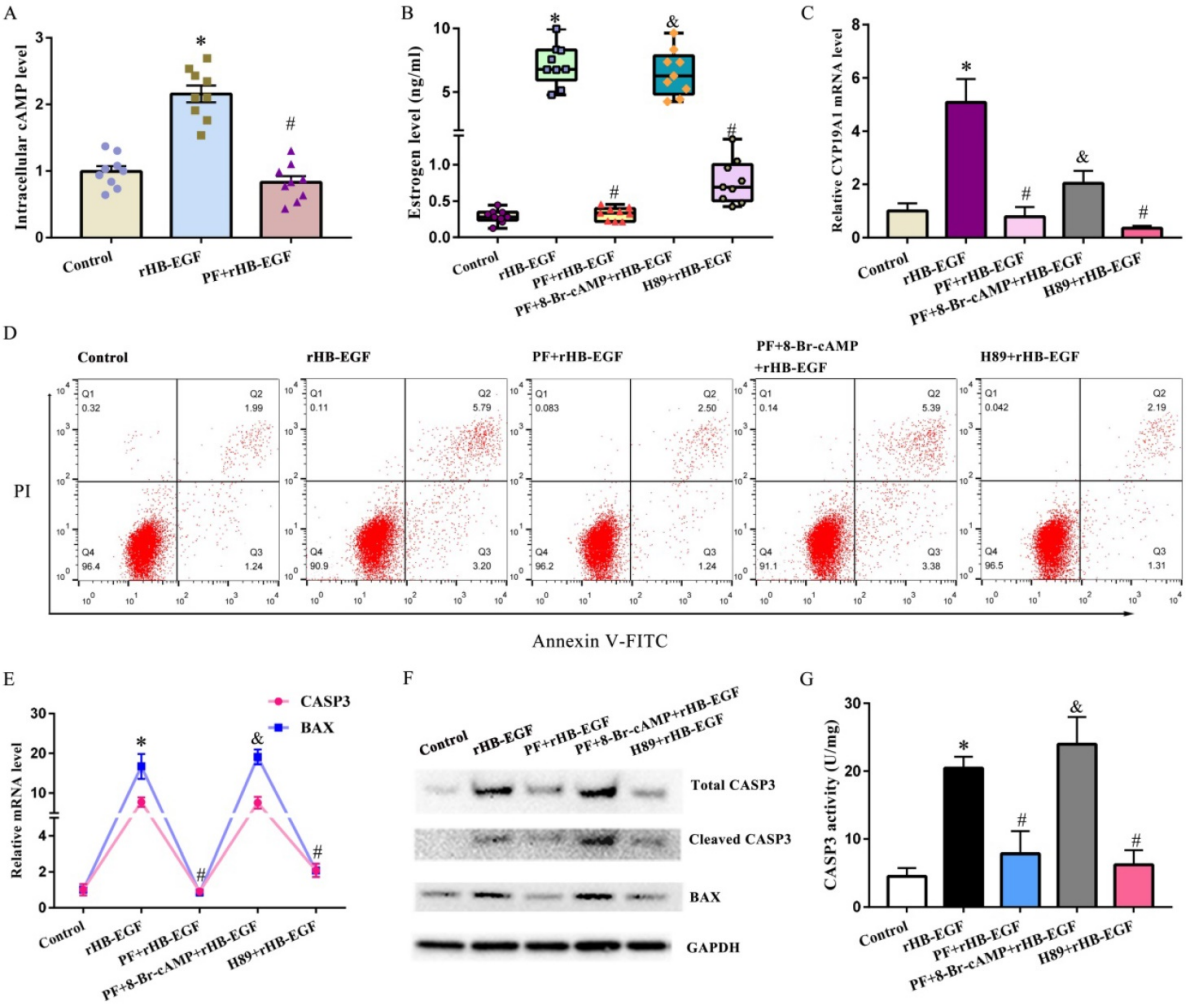
** cAMP-PKA signaling mediates the effects of HB-EGF on estrogen secretion and GCs apoptosis. A,** HB-EGF induced the accumulation of intracellular cAMP via EGFR. **B and C,** cAMP-PKA signaling mediated the effect of HB-EGF on estrogen secretion (N = 9) and CYP19A1 expression (N = 4). **D,** cAMP-PKA signaling mediated the effect of HB-EGF on GCs apoptosis. N = 3. **E-G,** Regulation of HB-EGF on the expression or activity of CASP3 and BAX was mediated by cAMP-PKA signaling. N = 3. ^*^ P < 0.05 versus control, ^#^ P < 0.05 versus rHB-EGF treatment, ^&^ P < 0.05 versus rHB-EGF plus PF299804 treatment.

In KGN GCs, HB-EGF induced the accumulation of intracellular cAMP, which was an important second messenger and principally activated PKA, but this induction was blocked by EGFR inhibitor PF299804 (Figure 2A). Replenishment of 8-Br-cAMP reversed the blockade of PF299804 on estrogen production and CYP19A1 expression, counteracted the rescue of PF299804 on GCs apoptosis, and antagonized PF299804 regulation of BAX and CASP3 expression as well as CASP3 activity under the context of rHB-EGF (Figure 2B-G). Concurrently, addition of PKA inhibitor H89 abrogated the induction of HB-EGF on estrogen secretion and CYP19A1 expression, impeded the apoptosis of GCs by HB-EGF, and lessened the expression or activity of BAX and CASP3 (Figure 2B-G).

### cAMP-PKA signaling mediates the regulation of HB-EGF on JNK and ERK

**Figure 3:**
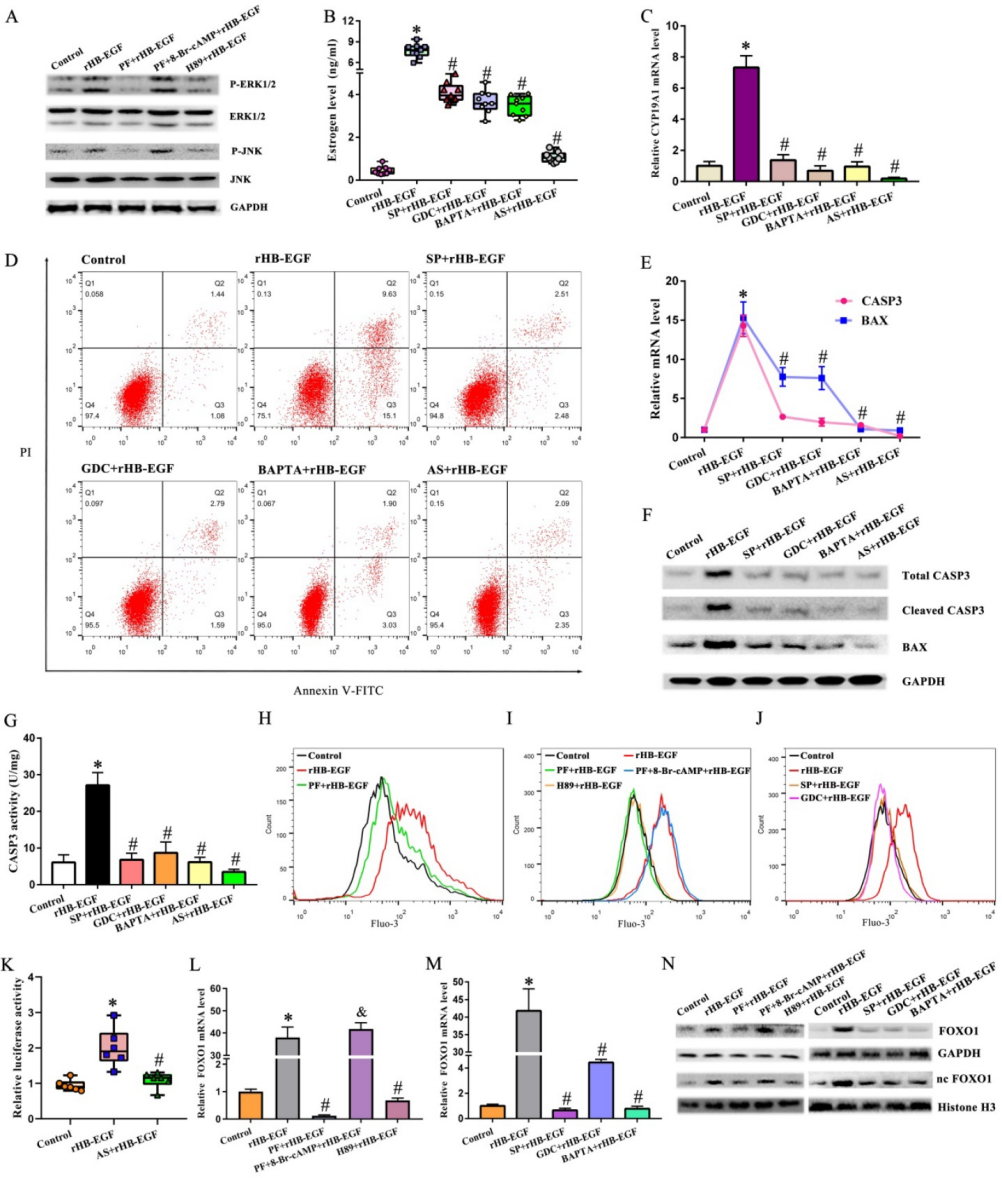
** HB-EGF induces estrogen secretion and GCs apoptosis via cAMP-PKA-JNK/ERK-Ca^2+^-FOXO1 pathway. A,** HB-EGF enhanced the expression of JNK and ERK via cAMP-PKA signaling. N = 3. **B and C,** HB-EGF induced estrogen secretion (N = 9) and CYP19A1 expression (N = 4) via JNK/ERK-Ca^2+^-FOXO1 pathway. SP, SP600125; GDC, GDC-0994; BAPTA, BAPTA-AM; AS, AS1842856. **D,** HB-EGF induced GCs apoptosis via JNK/ERK-Ca^2+^-FOXO1 pathway. N = 3. **E-G,** HB-EGF raised the expression or activity of CASP3 and BAX through JNK/ERK-Ca^2+^-FOXO1 pathway. N = 3. **H-J,** HB-EGF enhanced intracellular Ca^2+^ content via cAMP-PKA-ERK/JNK pathway. N = 3. **K,** Luciferase activity was assessed after GCs were transfected with pGL6-CYP19A1 plasmid and then treated with rHB-EGF in the absence or presence of FOXO1 inhibitor AS1842856. N = 6. **L-N,** HB-EGF increased FOXO1 expression via cAMP-PKA- JNK/ERK-Ca^2+^ pathway. N = 3.

Treatment of GCs with rHB-EGF resulted in the dramatic up-regulation for JNK and ERK phosphorylation, but this up-regulation was hampered by EGFR inhibitor PF299804 (Figure 3A). Further analysis evidenced that JNK inhibitor SP600125 and ERK inhibitor GDC-0994 hindered the stimulation of HB-EGF on estrogen level and CYP19A1 expression, alleviated the apoptosis of GCs conferred by HB-EGF, and restrained the expression or activity of BAX and CASP3 (Figure 3B-G). We next clarified whether cAMP-PKA signaling might mediate the regulation of HB-EGF on JNK and ERK. Replenishment of 8-Br-cAMP restored the induction of HB-EGF on JNK and ERK phosphorylation in the presence of PF299804, whereas PKA inhibitor H89 disrupted this induction of JNK and ERK phosphorylation by HB-EGF (Figure 3A).

### HB-EGF induces intracellular Ca^2+^ via cAMP-PKA-JNK/ERK pathway

After exposure to rHB-EGF, intracellular Ca^2+^ content was obviously enhanced, but this enhancement was prevented by EGFR inhibitor PF299804 (Figure 3H). Administration of intracellular Ca^2+^ chelator BAPTA-AM retarded the elevation of estrogen level and cell apoptosis rate along with an apparent decline for the expression or activity of CYP19A1, BAX and CASP3 in rHB-EGF treated GCs (Figure 3B-G). We next dissected the involvement of cAMP-PKA-JNK/ERK pathway in the regulation of HB-EGF on Ca^2+^. Blockade of PKA, JNK and ERK by the corresponding inhibitor brought about an inability of HB-EGF in facilitating the increase of intracellular Ca^2+^ content, whereas 8-Br-cAMP neutralized the resistance of PF299804 to Ca^2+^ content elicited by rHB-EGF (Figure 3I and J).

### HB-EGF increases FOXO1 expression via cAMP-PKA-JNK/ERK-Ca^2+^ pathway

After addition of rHB-EGF, GCs exhibited an obvious increase for FOXO1 mRNA and total protein as well as nuclear protein, while EGFR inhibitor PF299804 attenuated this increase (Figure 3L and N). Repression of FOXO1 by AS1842856 resulted in the defective capability of HB-EGF in inducing estrogen production and GCs apoptosis, and renewing the expression or activity of CYP19A1, BAX and CASP3 (Figure 3B-G). By bioinformatic analysis, CYP19A1 promoter region displayed the presence of FOXO1 binding site from +29 to +35. After transfection with CYP19A1-PGL6 plasmid, HB-EGF obviously enhanced luciferase activity, but this enhancement was abrogated by FOXO1 inhibitor AS1842856 (Figure 3K). We subsequently determined whether cAMP-PKA-JNK/ERK -Ca^2+^ pathway was implicated in the regulation of HB-EGF on FOXO1. Supplementation of corresponding inhibitor for PKA, JNK and ERK or addition of intracellular Ca^2+^ chelator antagonized the stimulation of HB-EGF on FOXO1 expression, while 8-Br-cAMP counteracted the improvement of PF299804 on FOXO1 expression in rHB-EGF-treated GCs (Figure 3L-N).

### HB-EGF causes mitochondrial dysfunction via cAMP-PKA-JNK/ERK-Ca^2+^-FOXO1 pathway

**Figure 4:**
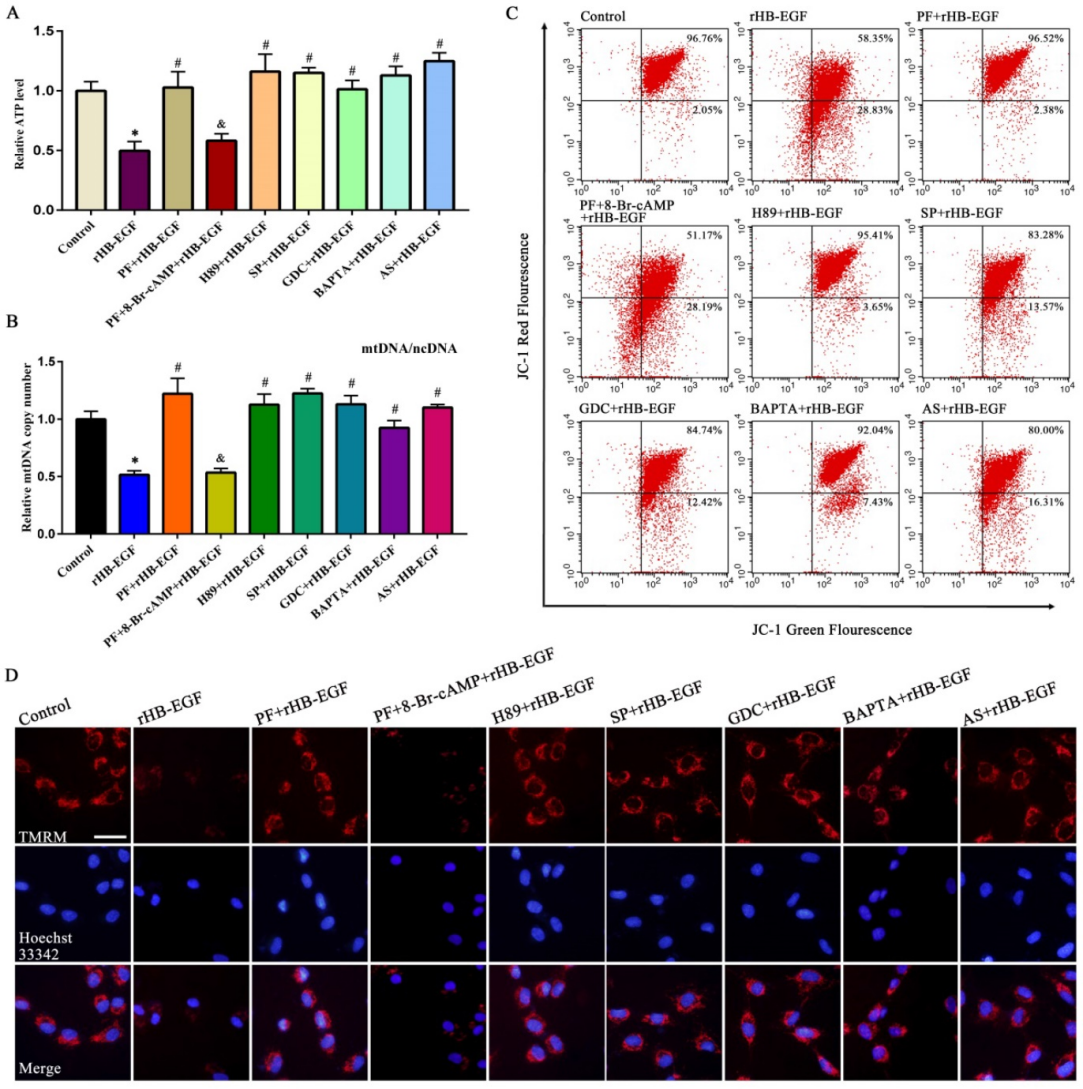
**HB-EGF causes the aberration of ATP level, mtDNA copy number and MMP via cAMP-PKA-JNK/ERK-Ca^2+^-FOXO1 pathway.** A and B, HB-EGF brought about the reduction of ATP content (N = 6) and mtDNA copy number (N = 5) via cAMP-PKA-JNK/ERK-Ca^2+^-FOXO1 pathway. C and D, HB-EGF attenuated the MMP via cAMP-PKA-JNK/ERK-Ca^2+^-FOXO1 pathway by flow cytometry analysis or visualization in fluorescence microscope. N = 3. Scale bar, 20 µm.

In KGN GCs, HB-EGF brought about the obvious reduction for ATP content and mtDNA copy number, and weakened MMP as indicated by a significant decline for red/green fluorescence intensity ratio (Figure 4A-C). To visualize MMP change, another MMP indicator was used. After treatment with rHB-EGF, fluorescence intensity of TMRM was attenuated (Figure 4D). Further analysis evidenced that blockade of PKA, JNK, ERK and FOXO1 by corresponding inhibitor or replenishment of intracellular Ca^2+^ chelator antagonized the diminishment of ATP content, mtDNA copy number and MMP conferred by HB-EGF, while 8-Br-cAMP resisted the rescue of PF299804 on aforementioned mitochondrial parameters in the existence of rHB-EGF (Figure 4A-D).

**Figure 5:**
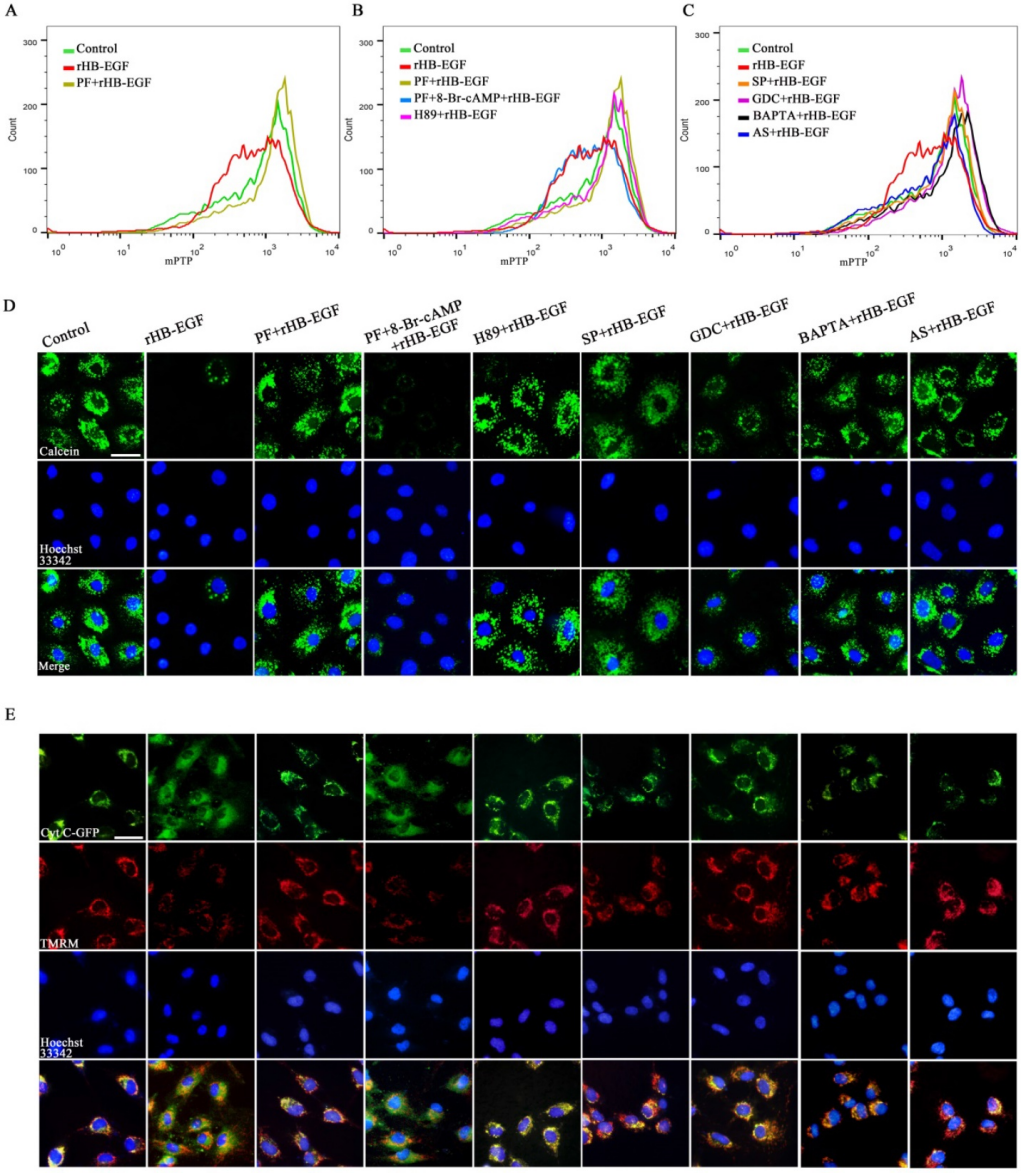
**HB-EGF induces mPTP opening and cytochrome C release via cAMP-PKA-JNK/ERK-Ca^2+^-FOXO1 pathway. A-C,** Flow cytometry analysis evidenced that HB-EGF induced the opening of mPTP via cAMP-PKA-JNK/ERK-Ca^2+^-FOXO1 pathway. N = 3. **D,** Visualization of mPTP after treatment with rHB-EGF in the absence or presence of different inhibitor or 8-Br-cAMP. N = 3. **E,** HB-EGF induced the release of cytochrome C via cAMP-PKA-JNK/ERK-Ca^2+^-FOXO1 pathway. N = 3.

**Figure 6:**
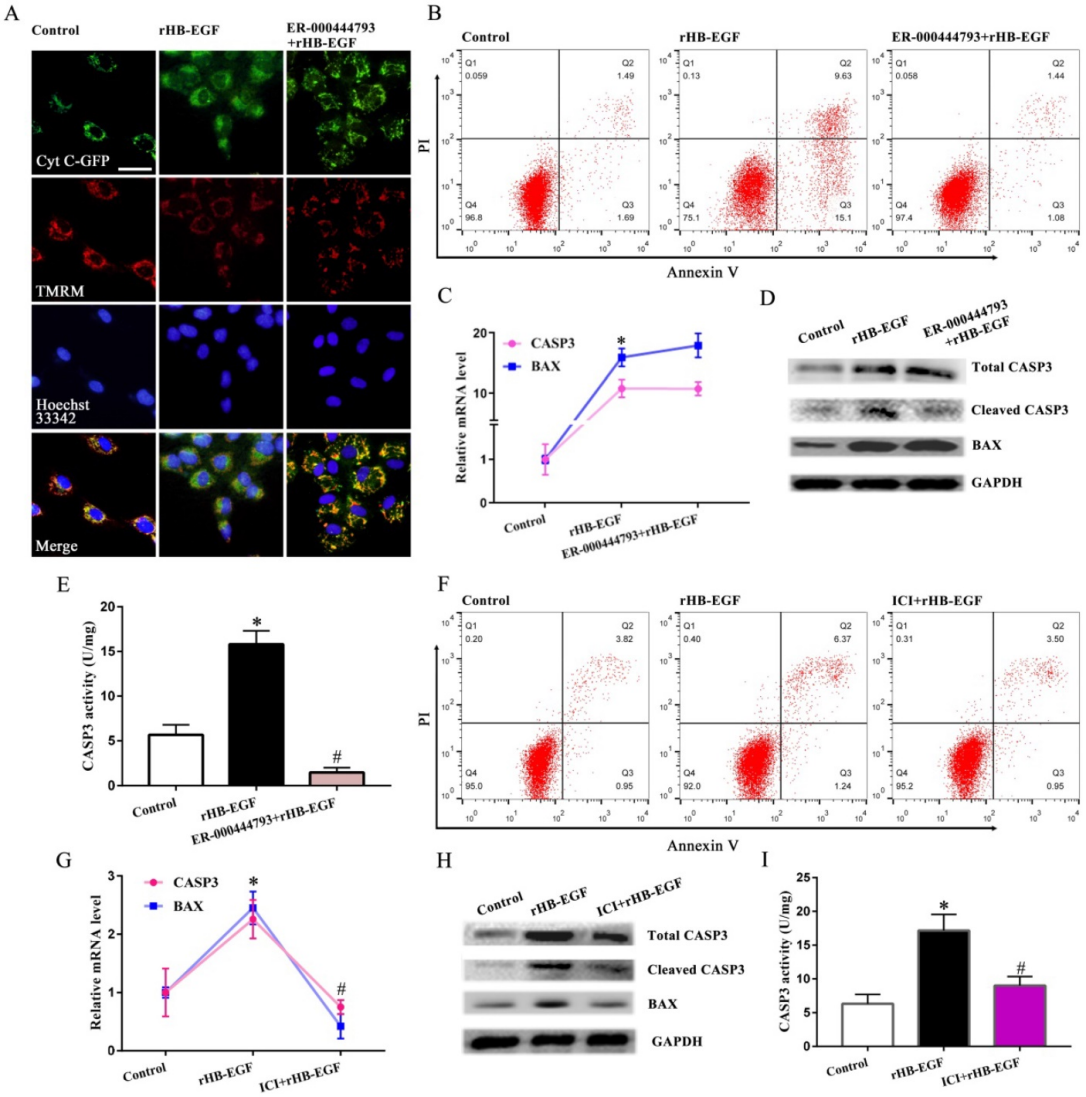
** HB-EGF induces GCs apoptosis through promoting mPTP opening and enhancing estrogen secretion. A and B,** mPTP opening inhibitor ER-000444793 hampered the induction of HB-EGF on cytochrome C release and GCs apoptosis. N = 3. **C-E,** Blockage of mPTP opening weakened the induction of HB-EGF on cleaved CASP3 expression and activity, while did not alter change its regulation on BAX. N = 4. **F,** ER antagonist ICI 182780 impeded GCs apoptosis by HB-EGF. N = 3. ICI, ICI 182780. **G-I,** ER antagonist ICI 182780 attenuated the facilitation of HB-EGF on the expression or activity of CASP3 and BAX. N = 3.

To further assess the role of HB-EGF in maintaining mitochondrial function, we analyzed its effect on mPTP opening and cytochrome C release. In GCs, HB-EGF induced the opening of mPTP as evidenced by the diminished fluorescence intensity of mitochondrial calcein and provoked the release of cytochrome C from mitochondria into the cytosol, whereas addition of EGFR inhibitor PF299804 counteracted above effectiveness, but this counteraction was of no avail after supplementation of 8-Br-cAMP (Figure 5A-E). Impediment of PKA, JNK, ERK and FOXO1 by corresponding inhibitor or adjunction of intracellular Ca^2+^ chelator opposed the promotion of HB-EGF on mPTP opening and cytochrome C release (Figure 5A-E). Further analysis demonstrated that after treatment with ER-000444793, a blocker of mPTP opening, release of cytochrome C was obstructed and GCs apoptosis rate was mitigated concomitant with the reduction for cleaved CASP3 expression and activity but not BAX (Figure 6A-E).

### HB-EGF induced GCs apoptosis and mitochondrial dysfunction via estrogen hypersecretion

**Figure 7:**
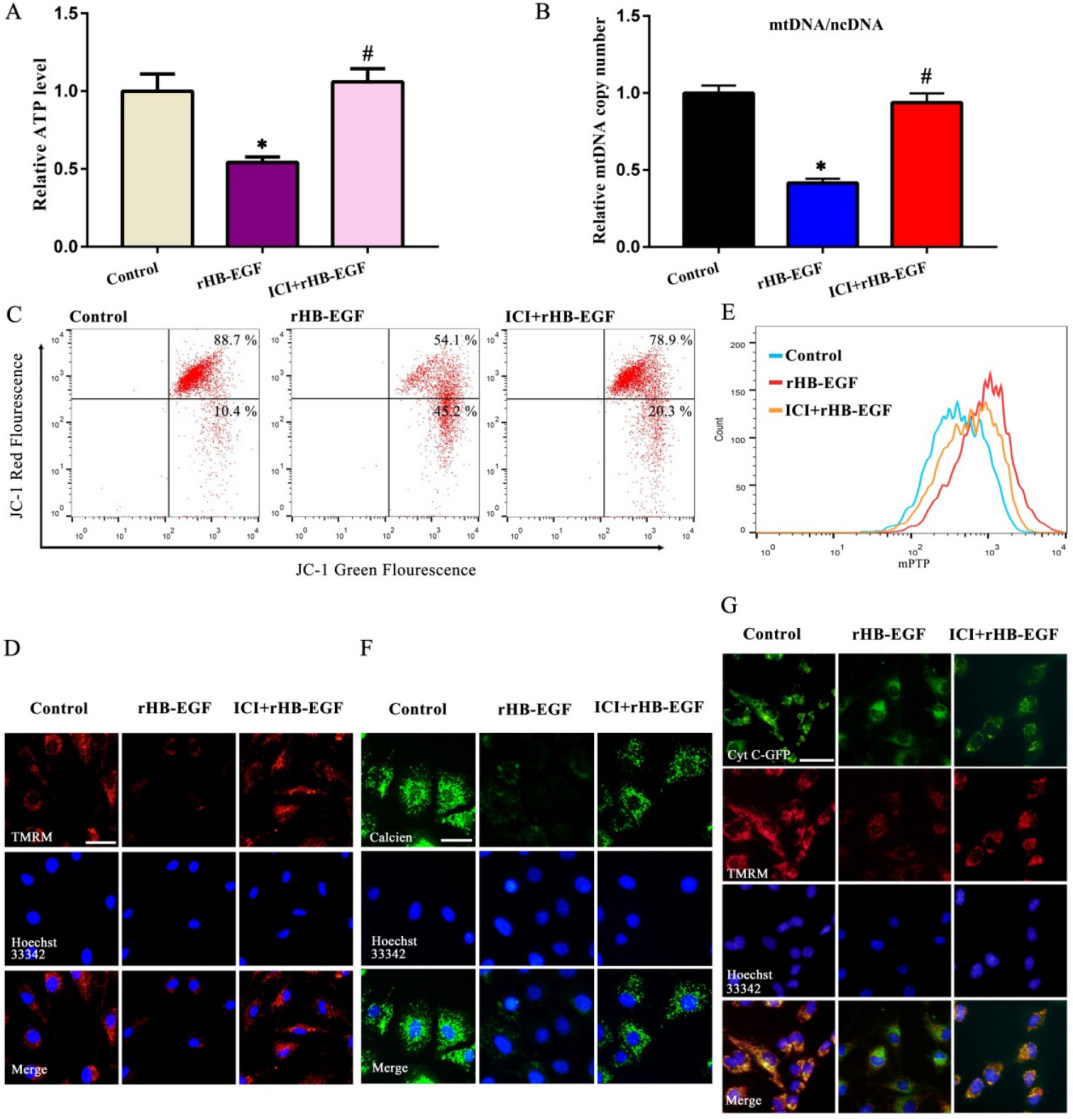
** HB-EGF impairs mitochondrial function through enhancing estrogen secretion. A and B,** Estrogen receptor antagonist ICI 182780 prevented the impairment of HB-EGF on ATP level and mtDNA copy number. N = 5. **C and D,** ICI 182780 resisted the regulation of HB-EGF on MMP. N = 3. **E and F,** ICI 182780 counteracted the induction of HB-EGF on mPTP opening. N = 3. **G,** ICI 182780 impeded the induction of HB-EGF on cytochrome C release. N = 3.

As described above, HB-EGF induced the hypersecretion of estrogen and enhanced GCs apoptosis. We next explored whether estrogen might mediate the effects of HB-EGF on GCs apoptosis. After exposure to estrogen receptor antagonist ICI 182780, HB-EGF presented the defective ability in inducing GCs apoptosis and enhancing the expression of CASP3 and BAX as well as CASP3 activity (Figure 6F-I). Furthermore, treatment of GCs with ICI 182780 brought about the apparent amelioration for aberrant ATP level, mtDNA copy number and MMP elicited by HB-EGF, hindered the opening of mPTP and impeded the release of cytochrome C from mitochondria into the cytosol (Figure 7A-G).

## Discussion

HB-EGF is implicated in the regulation of ovarian cancer progression, but its relevance with PCOS remains unknown. The present study exhibited the elevation of HB-EGF level in follicular fluid from PCOS women. Meanwhile, aberrant HB-EGF expression was also noted in PCOS patient GCs 18. Together these observations imply a potential involvement of HB-EGF in PCOS etiology. Ovarian GCs were required for folliculogenesis and ovulation, and its dysfunction was regarded as a predisposition of PCOS 3,4,19. HB-EGF induced the excessive production of estrogen, which was also noted in GCs from PCOS patients 5-7. Injection of estrogen into female mice brought about anovulatory and follicular cysts, while treatment with estrogen antagonist clomiphene citrate enhanced the ovulation rate of PCOS patients 8,20,21. CYP19A1 was an important enzyme in converting testosterone to estrogen and its inhibitor was used to treat anovulatory PCOS 21-23. In GCs, HB-EGF induced the expression of CYP19A1, further reinforcing the importance of HB-EGF in the regulation of estrogen synthesis. Further analysis evidenced that HB-EGF might exert its biology function via binding EGFR 24. Blockade of EGFR by PF299804 abrogated the induction of HB-EGF on estrogen production and CYP19A1 expression, indicating that EGFR was prerequisite for HB-EGF in the induction of GCs dysfunction.

As an important second messenger, cAMP was abundantly accumulated in GCs from PCOS women 6,25. In GCs, HB-EGF via EGFR induced the elevation of intracellular cAMP level which might principally activate PKA and its accumulation prevented oocytes from maturation 26,27. Treatment with PKA inhibitor H89 hampered the induction of HB-EGF on the granulosa production of estrogen, while replenishment of cAMP analogue 8-Br-cAMP counteracted the improvement of PF299804 on estrogen secretion under the context of rHB-EGF. Together these observations indicate that HB-EGF may transmit intracellular downstream signaling via cAMP-PKA pathway after binding to EGFR. Further analysis found that cAMP-PKA signaling mediated the regulation of HB-EGF on JNK and ERK which were important for folliculogenesis as well as ovulation, and their aberrant expression was also observed in PCOS 28-33. Addition of corresponding inhibitor for ERK and JNK disrupted the inducement of HB-EGF on estrogen secretion. Consistently, blockade of JNK attenuated the HB-EGF-induced cytotrophoblast cell migration, while repression of ERK weakened the DNA synthesis of vascular smooth muscle cells and intestinal restitution conferred by HB-EGF 34-36. Collectively, these data state that HB-EGF modulates the secretory function of GCs via JNK and ERK dependent on cAMP-PKA signaling.

Ca^2+^ was a versatile messenger molecule that operates numerous different cellular functions including hormone secretion and was crucial for oocyte maturation 37,38. Addition of intracellular Ca^2+^ chelator BAPTA-AM retarded the granulosa production of estrogen after different stimulation 39. In GCs, HB-EGF induced the elevation of intracellular Ca^2+^ content which was noted in the serum from PCOS patients 40 and its reduction by chelator BAPTA-AM hampered the effect of HB-EGF on estrogen secretion. Simultaneously, Ca^2+^ actuated the transcription of downstream target gene 41. Under the context of rHB-EGF, BAPTA-AM suppressed the expression of FOXO1 which was an important transcription factor in modulating follicular development and also involved in the pathogenesis of PCOS due to its effects on insulin resistance and chronic inflammation that were important features for PCOS 2,42-44. Repression of FOXO1 by AS1842856 alleviated the induction of HB-EGF on estrogen production. Taken together, these observations suggest that FOXO1 may serve as a downstream target of Ca^2+^ to mediate the regulation of HB-EGF on estrogen. Further analysis evidenced that blockage of JNK and ERK abrogated the stimulation of HB-EGF on intracellular Ca^2+^ accumulation and FOXO1 expression, implying that JNK and ERK exerts an important action in the crosstalk between HB-EGF and Ca^2+^ as well as FOXO1.

Mitochondrial dysfunction was considered as a crucial causative factor to PCOS aetiology and its improvement by mitochondria-targeted antioxidant MitoQ_10_ mitigated the symptoms of PCOS rats 45-47. In GCs, HB-EGF induced mitochondrial dysfunction as evinced by the reduction of ATP content, mtDNA copy number and MMP, but this dysfunction was ameliorated by estrogen receptor antagonist ICI 182780. Concurrently, mPTP definitely reflects the integrity of mitochondrial function 48. Treatment of GCs with HB-EGF resulted in the opening of mPTP which gave rise to the release of cytochrome C from mitochondria into the cytosol 48,49. BAX, an important gatekeeper of mPTP, was required for cytochrome C release 50. HB-EGF promoted the opening of mPTP and raised the expression of BAX, whereas addition of ICI 182780 resisted this effectiveness. Collectively, these evidences reveal that HB-EGF may impair mitochondrial function through enhancing estrogen secretion. Furthermore, following the release of cytochrome C, caspase-dependent cell death was triggered 49. HB-EGF enhanced the apoptosis rate of GCs, which might facilitate the abnormalities of folliculogenesis and account for anovulation and aberrant steroidogenesis in PCOS 51,52, and promoted the cleavage and activity of CASP3 that were the principal executor of apoptosis 49, while blockage of mPTP opening by ER-000444793 attenuated the induction of HB-EGF on GCs apoptosis, implying that HB-EGF induced apoptosis via the mitochondria-dependent pathway.

## Conclusions

**Figure 8:**
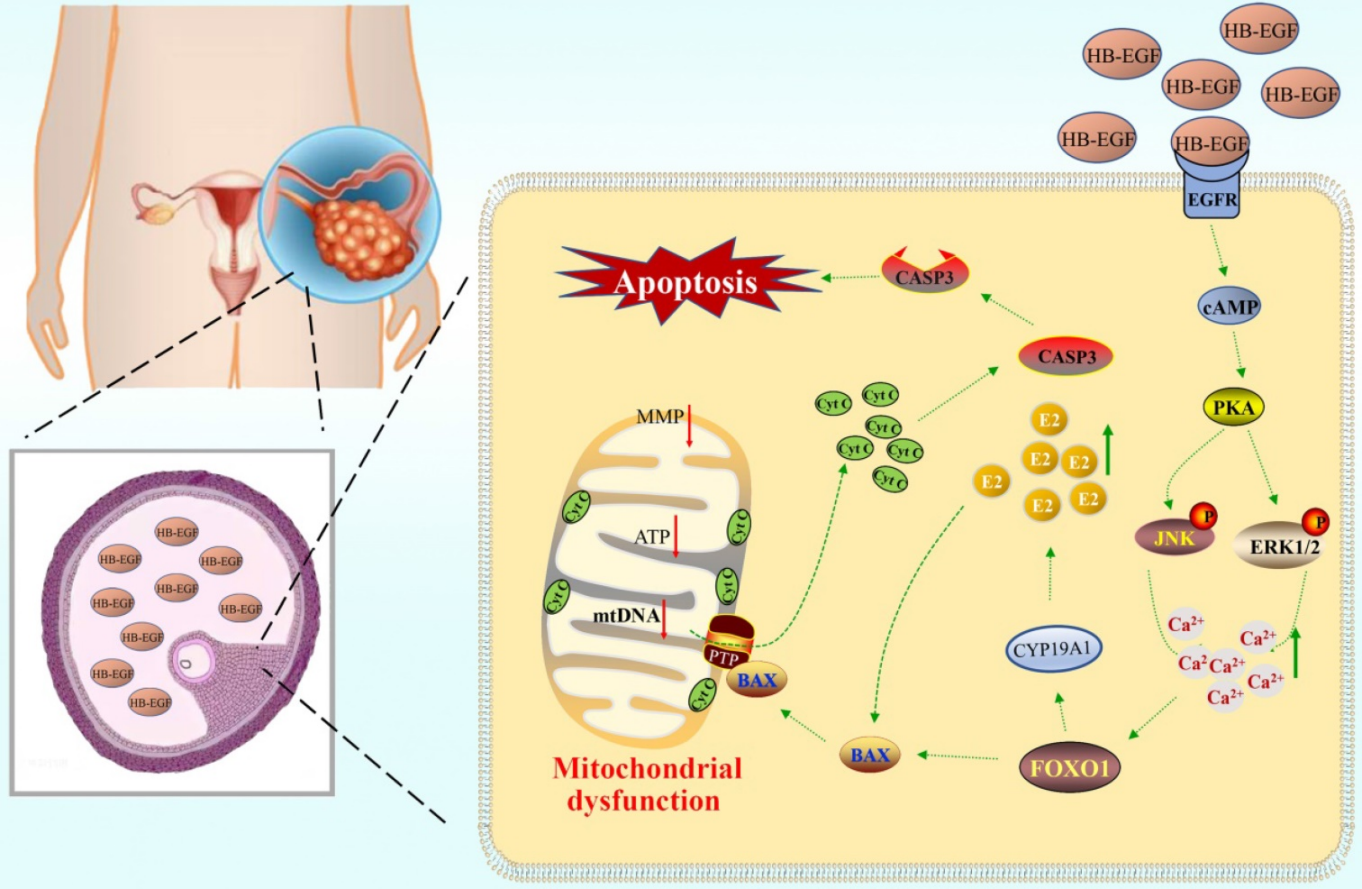
** Schematic depiction of HB-EGF regulation to GCs.** Elevated HB-EGF was noted in follicular fluid of PCOS patients, where it might induce the GCs production of more estrogen through cAMP-PKA-JNK/ERK-Ca^2+^-FOXO1 pathway after binding to EGFR and brought about mitochondrial dysfunction, resulting in the release of cytochrome C from mitochondria into the cytosol to trigger GCs apoptosis.

HB-EGF was abundantly noted in follicular fluid of PCOS patients, where it might bind to EGFR and induce the GCs production of more estrogen through cAMP-PKA-JNK/ERK-Ca^2+^-FOXO1 pathway, resulting in mitochondrial dysfunction and GCs apoptosis (Figure 8).

## Figures and Tables

**Table 1:** Primers used in this study

Gene	Primer Sequence	Accession number	Size
CYP19A1	GGACCCCTCATCTCCCACG CCCAAGTTTGCTGCCGAAT	NM_000103	195 bp
CASP3	CTGGACTGTGGCATTGAGAC GCAAAGGGACTGGATGAACC	NM_004346	159 bp
BAX	ACGGCCTCCTCTCCTACTTT GCCTCAGCCCATCTTCTTCC	NM_138761	107 bp
FOXO1	GAAGAGCGTGCCCTACTTCAA GATTGAGCATCCACCAAGAACT	NM_002015	149 bp
GAPDH	ATTTGGCTACAGCAACAGG TTGAGCACAGGGTACTTTATT	NM_002046	256 bp
